# Challenges and opportunities for novel combination therapies in Alzheimer's disease: a report from the EU/US CTAD Task Force

**DOI:** 10.1016/j.tjpad.2025.100163

**Published:** 2025-04-18

**Authors:** D. Angioni, L. Middleton, R. Bateman, P. Aisen, A. Boxer, S. Sha, J. Zhou, I. Gerlach, R. Raman, H. Fillit, S. Salloway, R. Sperling, B. Vellas, J. Cummings

**Affiliations:** a Institut Hospitalo Universitaire HealthAge, Alzheimer's Disease Research and Clinical Center, Toulouse University Hospital, Toulouse, France; b Ageing Epidemiology (AGE) Research Unit, School of Public Health, Imperial College London, London, UK; c Washington University School of Medicine, St Louis, USA; d USC Alzheimer's Therapeutic Research Institute, San Diego, California, USA; e Endowed Professor in Memory and Aging, Dept of Neurology, University of California, Memory and Aging Center, San Francisco, USA; f Neurology and Neurological Sciences Stanford Center for Memory Disorders, Stanford University, Palo Alto, USA; g Eisai Inc, Nutley, USA; h F. Hoffmann-La Roche Ltd, Basel, Switzerland; i Therapeutic Research Institute (ATRI), Keck School of Medicine of USC, University of Southern California, San Diego, USA; j The Alzheimer's Drug Discovery Foundation, New York, USA; k The Warren Alpert Medical School of Brown University, Providence, USA; l Brigham and Women's Hospital Massachusetts General Hospital, Boston, USA; m Science, Dept of Brain Health, University of Nevada Las Vegas (UNLV), Las Vegas, USA

**Keywords:** Amyloid, Tau, Clinical trials, Combination

## Abstract

Following the recent approvals of anti-amyloid immunotherapies as “first-in-kind” disease-modifying agents for Alzheimer's disease (AD), there is an emerging emphasis in combination therapies, given the complex and multifactorial etiopathogenesis and pathophysiology of the disease. The EU/US CTAD Task Force met in Madrid in October 2024, to discuss biological rationale and methodological issues and outline potential directions for future research in combination therapies. The Task Force agreed on the necessity and urgency of advancing combination therapies for AD treatment. As of January 1, 2024, in the drug development pipeline, there were 21 combination trials (13 % of all trials). The combination of anti-amyloid and anti-tau therapies could become a central focus of the field. Combinations involving anti-inflammatory and immune mechanisms with anti-amyloid or other therapies also have promise. To facilitate the development and implementation of combination therapies, collaborations between sponsors and public-private partnerships are essential. Optimizing the likelihood of success primarily requires leveraging the use of biomarkers and a clearer understanding of the biological mechanisms underpinning AD and their interactions, especially those involving amyloid, tau, and inflammation, that lead to cognitive decline and progression.

## 1 Introduction

Evidence from non-clinical research, observational biomarker-enriched studies, and autosomal dominant Alzheimer's Disease (ADAD) research converge on amyloid accumulation as a central driver of AD etiopathogenesis [1]. Recent trials have shown that cerebral amyloid plaque clearance is achievable, and this has been associated with a measurable slowing of clinical progression [2,3]. However, critical questions remain regarding the magnitude of these results, underscoring the unmet needs in this field and the urgent necessity of evaluating new strategies to achieve greater efficacy in future trials [4].

**Fig. 1:**
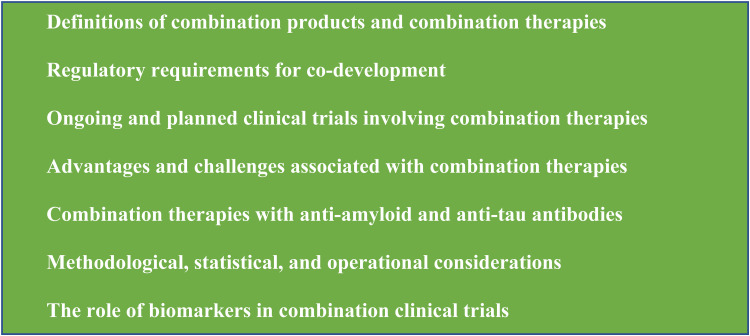
Key points addressed by the EU/US CTAD task force.

The complex and multifactorial nature of AD pathogenesis highlights the need for a comprehensive approach that engages multiple targets [5]. Lessons learned from other disease areas, such as cardiovascular diseases, cancer, metabolic diseases and human immunodeficiency virus (HIV), suggest that combining two or more drugs can maximize both biological and clinical benefits. Combination therapies may offer greater efficacy while minimizing the impact of dose limitations due to adverse effects [6]. However, various aspects of combination therapy development, ranging from biological rationale to methodological, statistical, and operational issues, require further consideration. The EU/US CTAD Task Force met in Madrid on October 29 to discuss these issues. This manuscript provides a summary of presentations from representatives of academia, industry, and regulatory bodies, along with the general discussions, while also emphasizing existing knowledge gaps and outlining potential directions for future research (Fig. 1).

## 2 Combination therapy development

### 2.1 Combination products and combination therapies

Combination therapies refer to the concurrent use of multiple treatments and do not necessarily require a single, unified product [7]. Combination therapies include:

• Approved drugs used together to enhance efficacy or reduce side effects
• One (or more) approved therapies (device or drug) paired with one (or more) new therapeutics (chemical or biologic) in clinical trials
• Add-on therapies (i.e., a treatment added to an existing primary therapy to enhance its effectiveness)
• The combination of two new therapeutics [8].

Combination products are medical products that combine two or more of three different types of products: drugs, devices, and biologics (i.e., drug-biologic, device-biologic, drug-device or even all three - drug, device and biologic combinations) in a single product, developed together or as fixed combinations [8]. The US Food and Drug Administration (FDA) classifies combination products based on their packaging and labeling into:

• Single-Entity Products: two or more components that are combined into a single product (e.g., prefilled syringes, transdermal patches, or drug-coated stents)
• Co-Packaged Products: products packaged together as a kit, but which retain their individual regulatory identities (e.g., surgical kits, in which the components, are provided together but are not physically combined)
• Cross-Labeled Products: separate components that are labeled for use together (e.g., dedicated drug and a dedicated infusion pump).

The constituent parts refer to the individual medical products included within the combination [8]. In the European Union (UE), the terms of integral product or drug-device combination are used to refer to devices incorporating a medicinal substance and devices intended to administer a medicinal product [9].

### 2.2 Regulatory requirements for co-development

While the constituent parts retain their original regulatory status and requirements, the combination product itself is considered a unique product category and must meet specific regulatory guidelines. Regulatory requirements for combination products generally reflect the typical requirements for each constituent part of the combination, ensuring that all components meet safety, efficacy, and regulatory standards for their respective categories. Combination products are reviewed under premarket approval pathways for drugs, devices, and biologics [8]. The FDA states that these products are submitted under a single investigational or marketing application, simplifying the approval process. The jurisdiction for combination products may be under one of three centers: CDER (Center for Drug Evaluation and Research), CBER (Center for Biologics Evaluation and Research), or CDRH (Center for Devices and Radiological Health). The assignment of jurisdiction is based on the primary mode of action (PMOA) of the combination product, which refers to the means by which a product achieves its intended therapeutic effect. The PMOA is defined as the single mode of action that provides the most important therapeutic impact, i.e., the action expected to make the greatest contribution to the overall intended therapeutic outcome. In cases where the primary mode of action is not clear, jurisdiction is determined based on experience and expertise within the relevant regulatory body. In the EU, the Medical Device Regulation distinguishes between the 'principal' and 'ancillary' actions of the medicinal substance in relation to the device. Similar to the United States, the 'principal' mode of action is the key criterion used to determine how the product is regulated and by which regulatory authority [9].

Drug-drug combinations are a special category referred to as fixed combination prescription drugs or fixed dose combination drugs. The European Medicines Agency (EMA) provides guidelines on the co-development of fixed combination medicinal products. In this document, the EMA states that the primary scientific requirement for any fixed combination product is to justify the pharmacological and medical rationale behind the combination. The combined use of active substances should offer a superior benefit-risk profile by enhancing efficacy and/or improving safety compared to using any of the individual substances alone. It is essential to establish a robust evidence base that demonstrates the contribution of each active substance to the therapeutic effects and the overall positive benefit-risk profile of the combination for the intended indication [10]. The FDA states that drug-drug combinations are reviewed under « the combination rule » stating that two or more drugs can be combined in a single dosage form when each component contributes to the intended effects, and the dosage of each component (amount, frequency, duration) ensures that the combination is safe and effective [11]. Co-development may be less informative on the safety, effectiveness, and dose-response of the individual investigational drugs designed for combined use, compared to what would be obtained if the drugs were developed independently. As a result, this approach may present greater risks and should therefore be considered only in situations that meet specific criteria [12], including:

• A strong biological rationale supporting the combination
• The combination is developed to treat a serious disease
• Non-clinical or early clinical data suggest that the combination therapy is more effective than currently available treatments and superior to the individual components alone
• A strong justification for why the components cannot be developed separately.

### 2.3 Non-clinical and clinical phases of co-development

The development of combination therapies involves both non-clinical and clinical studies. The non-clinical phases include essential steps to ensure dosing, safety and scientific rationale for the combination. Safety requirements depend on the amount of clinical data already available for each individual component. When considering the co-development of two late-stage components (drugs with significant clinical experience from Phase III trials or post-marketing studies) or a late-stage with an early-stage component, additional toxicity studies are generally not required if sufficient clinical experience with the combination is available, and no safety concerns exist. In the absence of sufficient clinical experience with the combination and no specific safety issues of the constituent individual drugs, there is no requirement of non-clinical toxicity studies for small-scale, short-term trials of combination therapies. Non-clinical toxicity studies (duration of 90 days) become necessary to support larger or longer studies, marketing application, or when co-developing two early-stage agents in the absence of clinical experience with their combination. Clinical studies for co-development include early Phase I studies, clinical pharmacology and proof-of-concept studies (Phase II) and confirmatory studies (Phase III). Phase I studies are intended to explore safety parameters (e.g., Maximum Tolerated Dose, Dose-Limiting Toxicity), pharmacokinetics, and dose-response for available biomarkers to inform combination dosing in later-stage trials. In pharmacological studies, safety parameters and dose-response should be evaluated as if the different agents were developed separately, but also in the context of their combination, to identify any new interaction or altered behavior when the drugs are used together. Phase II should demonstrate (or further demonstrate) the contribution of each component, typically using a factorial design with possible adaptive features (likely based on validated biomarkers) to drop ineffective combinations, provide evidence of the combination's effectiveness, and identify the optimal dose to retain for Phase III. Phase III designs are typically tailored based on prior development stages. For instance, if the contribution of each component has been adequately established by earlier investigations, Phase III can simply compare the combination to the control [12].

### 2.4 Add-On studies

Add-on studies are a type of placebo-controlled study (or other control), in which the new treatment and placebo are each added to a common standard therapy resulting in a combination treatment. Add-on studies may or may not result in an adjunctive indication. In many cases, add-on studies do not raise development requirements for combination products designed to be used together [7]. This design is most useful in the following situations: a standard treatment is not fully effective, available treatment is known to decrease mortality or irreversible morbidity, a non-inferiority trial using the standard treatment as the active control may not be feasible or could lead to challenges in interpreting the results. These studies are most likely to succeed when the add-on and standard treatments have different pharmacologic mechanisms. Add-on studies provide evidence of improved clinical outcomes rather than merely demonstrating non-inferiority. Efficacy is established by such studies specifically for the combination treatment. For instance, considering a single agent, the dose found to be effective in a combination therapy might differ from that used as monotherapy.

## 3 State of the art of combination therapy trials FOR alzheimer's disease

### 3.1 Combination trials in the AD drug development pipeline

Combination therapies are classified into pharmacodynamic and pharmacokinetic combinations according to their mechanisms. Pharmacodynamic combinations involve two or more treatments with effects that may be either additive or synergistic. These combinations can include two or more drugs (i.e., approved products or new chemical entities) developed together or separately, a combination of drug(s) with other treatment modalities (e.g., devices or lifestyle interventions), natural combination therapies (e.g., traditional Chinese medicines, herbal formulations), or single agents encompassing two or more treatment effects (i.e., single agents with multiple targets affecting multiple disease-related pathways or mechanisms, or single agents with one target that affects multiple disease-related pathways or mechanisms). Pharmacodynamic combinations also encompass the sequential combination of two approved agents and the association of two or more drugs in a single pill [7]. Pharmacokinetic combinations involve an active agent paired with other treatment(s) designed to modulate its metabolism or reduce peripheral side effects [7].

As of January 1, 2024, there were 21 combination trials in the AD drug development pipeline, accounting for 13 % of all trials, including 11 pharmacokinetic combinations and 9 pharmacodynamic combinations (Table 1) [13]. All of these were drug-drug combinations, with no drug-device or drug-lifestyle combinations. The METFINGER trial combining structured lifestyle interventions plus metformin [14] was not specifically designed as an AD trial. Among the pharmacodynamic combinations, six combinations consist of multiple drugs targeting a single process (e.g., inflammation, senolytics, bioenergetics, gut-brain axis), while 4 combinations target multiple processes (e.g., tau + amyloid). Four of these combinations are “senolytic” agents. These combination trials involve repurposed agents, none of them include a new chemical entity. Eight combinations are being developed as two treatments administered together, with one configured as an add-on therapy (E2814) to lecanemab. All pharmacokinetic combination trials target neuropsychiatric symptoms. Two combinations entail blocking the peripheral metabolism of the active agent, while one focuses on blocking peripheral side effects. During the year 2024, three trials testing AVP-786 (dextromethorphan + quinidine) were halted due to lack of efficacy.

**Table 1:** Pharmacodynamic and Pharmacokinetic combination therapies in the 2024 drug development pipeline.

PHARMACODYNAMIC COMBINATIONS		
Agents	Targets	Number of Trials
Ciprofloxacin + Celecoxib	Neuroinflammation	1
Dasatinib + Quercetin	Senolytic	3
Lecanemab + E2814	Amyloid; Tau	2
Insulin + Empagliflozin	Bioenergetics	1
Resveratrol + Quercetin + Curcumin	Senolytic; Anti-inflammatory; Antioxidant	1
GV-971 + Memantine	Gut-brain axis	1
Wujia Yizi Traditional Chinese Medicine	Bioenergetics	1
Diamine Oxidase Inhibitor + Antioxidant	NMDA Receptors; Cognitive enhancer	1


PHARMACOKINETIC COMBINATIONS		
Agents	Targets	Number of Trials
Xanomeline + Trospium	AD psychosis	4
Dextromethorphan + Quinidine	AD agitation	3
Dextromethorphan + Bupropion	AD agitation	2

Adapted from J. Cummings presentation « Combination Therapies: An Overview of On-Going Trials » CTAD Task Force 2024.

### 3.2 Ongoing and planned clinical trials involving anti-amyloid therapies

Three clinical trials combine anti-amyloid and anti-tau therapies. The combination of lecanemab and E2814 is being evaluated in the Dominantly Inherited Alzheimer's Network (DIAN) Treatment Unit (DIAN-TU) NextGen trial (NCT01760005) [15]. E2814 is an IgG1 monoclonal antibody that selectively binds to the microtubule-binding region (MTBR) of tau that demonstrated robust target engagement in a Phase Ib trial, as well as a significant reduction in cerebrospinal fluid (CSF) microtubule binding region (MTBR)-tau243 and p-tau217 levels in DIAN-TU studies [16]. The DIAN-TU NextGen trial includes both symptomatic DIAD patients and asymptomatic mutation carriers as two separate cohorts. In the symptomatic cohort, lecanemab is initiated first in all patients, followed by randomization to E2814 or placebo (1:1) starting six months later, after the expected peak risk period of Amyloid-Related Imaging Abnormalities (ARIA) has lapsed. The primary outcome measure of the trial is the progression of tau during E2814 treatment, assessed using tau positron emission tomography (PET) imaging in the symptomatic cohort. In the asymptomatic cohort, patients are initially randomized into the E2814 or placebo arms before beginning lecanemab treatment after 12 months. In the asymptomatic population, the primary outcome measure is p-tau217/total tau ratio ( %ptau-217) in CSF. The study enrolled its first patient in January 2022 and will run through 2027.

A phase II dose-finding trial combining E2814 and lecanemab has recently been initiated in patients with early AD and confirmed amyloid and tau pathology (NCT06602258). All participants will receive weekly subcutaneous doses of lecanemab as background therapy, along with one of four dose levels of E2814 (or placebo) administered every four weeks for 18 months. The primary outcome measure is CSF MTBR-tau243 [17].

Another phase II combination trial planned to start soon is the Alzheimer's Tau Platform (ATP), which aims to evaluate the safety, tolerability, and effects on tau biomarkers of two distinct tau regimens in individual with preclinical and prodromal AD [18]. Each regimen includes three arms: an anti-amyloid therapy, an anti-tau therapy, and a combination of both. The ATP trial consists of an initial 24-month double-blind phase, followed by an open-label extension where participants receive the combination of anti-amyloid and anti-tau treatments. The primary outcome measure of the study is tau PET accumulation rate, measuring the progression of tau deposition over time.

Addressing the amyloid cascade from both ends, i.e., by reducing the production of toxic monomers and oligomers while simultaneously clearing aggregated forms, may ultimately lead to a stronger therapeutic effect. RG6289, a second-generation γ-secretase modulator, is being developed for the treatment of AD, with the aim of slowing amyloidogenesis. This drug demonstrated a dose-dependent reduction in Aβ42/40 and an increase in Aβ37/38 concentrations in the CSF of healthy volunteers in a Phase I trial [19]. The ongoing phase II study will provide further insights into the potential of RG6289. The Alzheimer's Prevention Initiative (API) – 2 study is currently designed to evaluate the concurrent and sequential combination of donanemab and RG6289 in a Colombian kindred with the Paisa presenilin-1 mutation. In the first stage of this trial, mutation carriers will be treated with donanemab. Amyloid PET will be conducted at baseline, 9 months, and 18 months. Once participants reach amyloid clearance (defined as an amyloid burden under 11 centiloids), they will be randomly assigned to one of the following four groups: RG6289, donanemab, combination of RG6289 and donanemab, or placebo. This design will inform changes in biomarkers and cognitive decline, as well as identifying the most effective treatment strategy for preventing plaque re-accumulation after amyloid clearance is achieved. The API-2 study would not be the first example of combination therapy involving Donanemab. The original design of the phase II TRAILBLAZER-ALZ trial (NCT03367403) included four arms, one of which consisted in the combination of Donanemab and LY3202626 a Beta-Site Amyloid Precursor Protein Cleaving Enzyme 1 (BACE1) inhibitor. However, due to adverse effects associated with BACE inhibitors, this arm was removed from the trial.

## 4 Key questions about combination therapies IN alzheimer's disease clinical trials

### 4.1 Advantages and challenges of combination therapies

Combination therapies for AD offer several potential advantages, beyond the previously cited biological and potential clinical benefits, by targeting multiple disease mechanisms. Firstly, this approach would deepen our understanding of the relationship between amyloid and tau pathology, as well as elucidate the impact of anti-amyloid antibodies on multiple tau mechanisms. Logistically, combination treatment trials can offer practical advantages, such as reduced number of participants required, and potentially lower costs compared to separate trials for each drug.

However, combination therapy approaches also present significant challenges. A major factor is the difficulty of distinguishing the safety and efficacy effects of the different drugs used in the combination, making it challenging to identify which drug is responsible for the observed effect. This could be addressed by implementing designs which have parallel monotherapy arms, e.g., factorial designs, or monotherapy followed by combination. One goal of combination treatment is to determine whether the combination results are additive, subtractive, or synergistic in their effects, as this information can impact treatment decisions and future drug development. Additional considerations include how to identify effective and safe dosing in combination, determine the appropriate frequency for assessing the effects of the combination, and evaluate each drug's impact on the stage of the disease (e.g., asymptomatic versus early symptomatic cohorts).

Combination trials present unique challenges, including trial design complexity, regulatory hurdles, and logistical demands. The assessments intended to evaluate safety and efficacy may vary depending on the agents considered. The combination of two (or more) drugs amplifies operational complexity and participant burden by requiring additional administration and safety assessments (e.g., magnetic resonance imaging (MRI) protocols for ARIA plus additional safety for tau drugs, combined administration protocols: drug A every 2 weeks, drug B every 4 weeks). Combination therapies may impose potentially longer and/or more frequent visits. Maintaining regulatory filings for two drugs (and associated procedures) can be challenging, depending on the development stage of each drug and their respective regulatory timelines, potentially delaying the ability to quickly modify or adapt a trial due to regulatory submission requirements of only one change at a time. Complexities may also arise due to the fact that different components of the combination therapy can be at different stages of market approval. Three different scenarios are possible:

• Premarket co-development model: none of the components in the combination therapy have yet been approved by regulatory authorities
• Post-market combination model: all drugs in the combination are already approved but have not been formally tested together for AD treatment
• Hybrid model (pre-market for one, and post-market for other): one drug is already approved, but the other is still in clinical development.

Various sponsorship models can be considered, and their applicability varies depending on the specific scenario. A joint venture approach involves two or more pharmaceutical companies coming together to create a temporary partnership or joint venture for the development and commercialization of the combination therapy. An independent trial sponsorship consists of an independent research institution being responsible for designing, conducting, and managing the trial. In a lead sponsor approach, the company developing the investigational drug assumes the leading role, establishing agreements with the manufacturer of the post-market drug for collaboration or data access. The costs, complexity, and operational demands of combination trials risk steering the field in a direction where only a small number of candidates can be tested by a limited number of extremely well-resourced sponsors. This seems counterproductive to the need to efficiently test large numbers of novel agents, particularly in an era where computational approaches and network pharmacology can help identify new, promising therapeutic strategies.

### 4.2 Which design should be chosen?

**Fig. 2:**
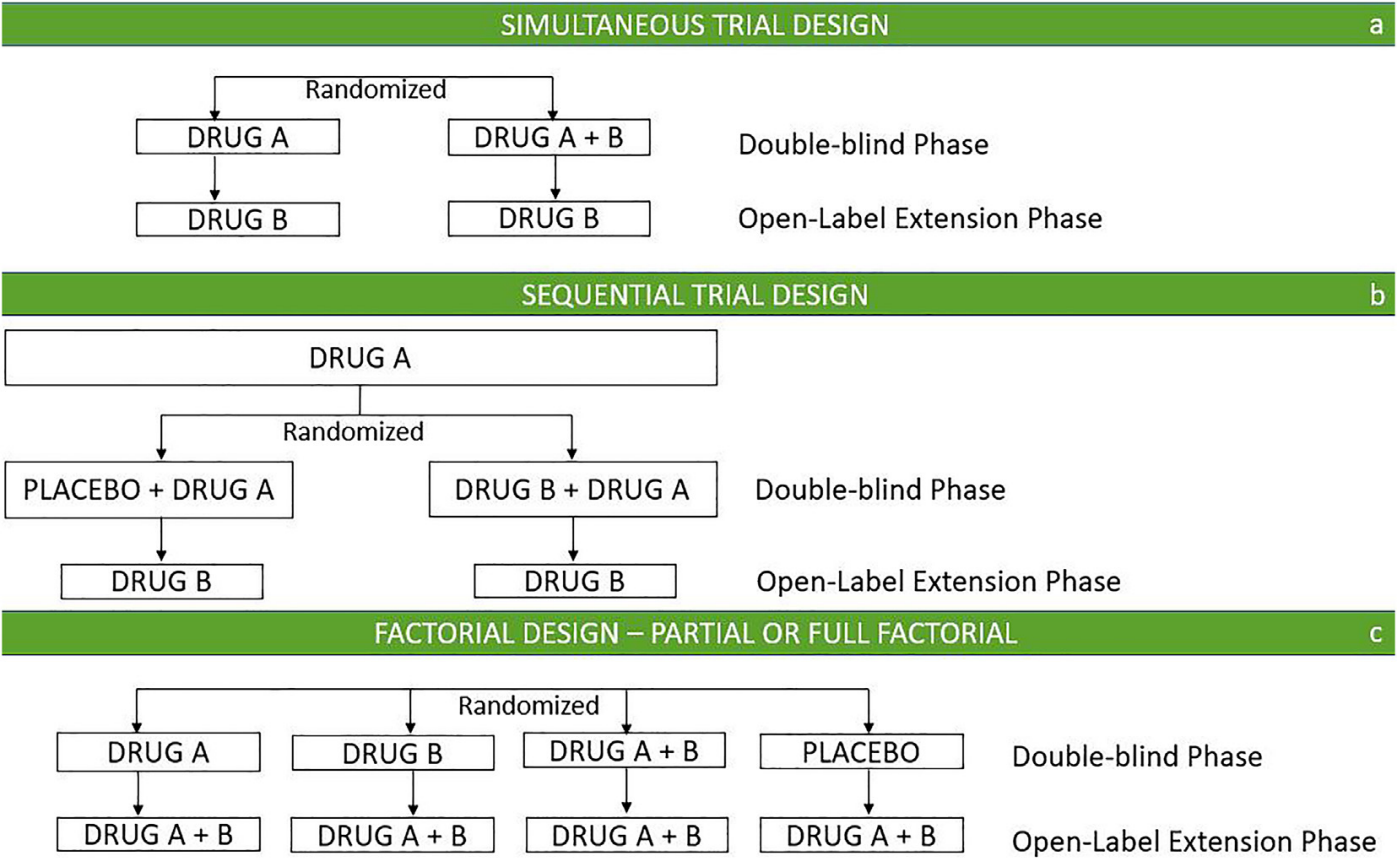
Combination therapy clinical trials designs. Adapted from R. Raman presentation « Statistical Issues in combination drug trials » CTAD Task Force 2024.

The simultaneous trial design (Fig. 2a) refers to a clinical trial model in which multiple interventions are evaluated in parallel. In this design, a double-blind phase is conducted to compare an approved drug in monotherapy (drug A) with a combination of this drug and a new investigational therapy, as an « add-on » arm (drug B). This first phase is usually followed by an open-label extension, in which all participants are treated with the new investigational drug. A key strength of this trial design is its requirement for a relatively small sample size with just two arms, while ensuring that all participants receive an active intervention. This approach facilitates recruitment and retention and closely mirrors real-world clinical practice. However, the lack of a monotherapy arm for the new investigational drug makes it challenging to differentiate between the individual effects of each drug and the effects of the combination therapy. Moreover, the absence of a monotherapy B arm did not allow for a direct comparison between the effects of drug A and drug B. For instance, if the combination of *A* + *B* appears more effective than A alone, it could erroneously lead to the approval of *A* + *B*, especially if B alone is the more effective drug and provides the same benefit when used alone as it does in combination with A. Additionally, the absence of a placebo-only arm prevents a comprehensive evaluation of the safety and efficacy of the component agents.

The sequential treatment trial design (Fig. 2b) refers to a clinical trial model in which multiple interventions are tested in successive stages. In the first stage, all patients receive the approved drug A. Once the safety period is completed, individuals are randomized to continue with either the placebo and the approved drug A, or the combination of approved and the new interventional drugs (*A* + *B*)**.** A third open-label extension (OLE) phase, in which all patients receive the new agent B, completes the trial. Alternatively, all patients could receive combination therapy in the OLE. Similarly to the simultaneous design, all participants receive an active intervention, and both interventions are studied simultaneously in the second phase of the trial. This design presents the limitations of the simultaneous design and additionally foresees a potentially longer duration and delays additional treatment when the second agent (e.g., anti-tau agent) may be beneficial earlier.

A factorial trial design (Fig. 2c) is used to simultaneously evaluate the effects of two or more interventions, typically examined separately, in combination, and against placebo. A partial factorial design refers to the exclusion of one of these arms. This trial design offers an optimal approach with direct comparisons of the combination against monotherapy and placebo, ultimately providing valuable insights into the different agents in terms of efficacy and safety. A major benefit of this design is that it enables the assessment of whether one monotherapy is more effective than the other. The main challenge lies in the presence of multiple arms, so all treatments proposed in monotherapy must be ethical and feasible for randomization. Additionally, participant perceptions and preferences can interfere with the randomization process, potentially impacting the study's ability to produce unbiased results. Finally, these trials tend to be more expensive, longer in duration, and larger in scale. To execute a factorial or a partial factorial trial design several key questions must be addressed to ensure feasibility and effectiveness. It is necessary to clearly define the research priorities (e.g. evaluating treatment effects, explore interaction between factors), identify the factors and their appropriate levels, and determine potential interactions. Sample size calculation is also important, as factorial designs can require large samples depending on the number of factors and levels. Power calculations are essential to ensure valid and reliable results. Data analysis methods must be defined, to interpret main effects and interactions. The design's feasibility should also be assessed, considering available resources, time, and budget. Finally, identifying potential limitations and trade-offs is important, especially when simplifying the design to make it more manageable while explaining the choices to stakeholders.

Various elements must be considered before selecting the most appropriate trial design. The trial goal should be clearly defined, particularly whether the priority is to assess the safety and efficacy of the new drug alone or in combination with a well-known agent. The need for a placebo or monotherapy arms requires consideration. The feasibility and ethics of randomization to monotherapy and/or placebo arms will depend on several factors, including the study population (asymptomatic vs. symptomatic) and the proposed interventions (early-stage agents vs. standard of care). The choice of the study design and analytic approaches should allow for a mechanistic understanding of each drug's contribution to the treatment effect. For biomarker-driven designs, inclusion of biomarkers to measure effects of each intervention (e.g., amyloid and tau) is desirable, assuming there is sufficient knowledge of each biomarker's performance, its clinical utility, and how it relates to other biomarkers in natural history studies. Finally, the impact of complex consent forms, participants' preferences for intervention arms, and the challenges in randomization and masking, especially in adaptive designs, must be carefully evaluated.

Clinical trial design should minimize the likelihood of false-positive findings (Type I error) while maximizing the ability to detect true treatment effects (statistical power). Inclusive recruitment goals and differences in access to amyloid-lowering therapies require an evaluation of the statistical operating characteristics. The sample size will be impacted by several elements, such as the heterogeneity of the study population, the expected treatment effect on different subpopulations, and the magnitude of the interaction effect between combination agents (i.e., additive, synergistic or antagonistic). For instance, assuming a small but important combination drug effect will increase the required sample size. Adaptive trial designs, utilizing both Bayesian and Frequentist approaches as appropriate and feasible, can facilitate more efficient trial evaluations, leading to earlier conclusions.

### 4.3 Should amyloid and tau therapies be used together?

The combination of anti-amyloid and anti-tau therapies has the potential to become a central focus soon. The biological rationale for combining anti-amyloid and anti-tau treatments arises from the evidence showing the common co-occurrence of these pathologies and observations supporting a mutual influence between the two pathways. On one hand, beta amyloid accumulation might act as the key driver of tau pathology. Indeed, oligomers and amyloid plaques, likely exert multiple effects on neurons, glial cells and vascular cells. These interactions contribute to cellular dysfunction and may promote the development and propagation of tau aggregates [20]. On the other hand, tau pathology may influence amyloid, potentially through a feed-forward mechanism involving promotion of amyloid release by soluble tau species. Studies examining many human brains throughout the lifespan reveal that tau pathology begins at least a decade prior to the formation of Aβ plaques [21,22]. In-vitro experiments with human-cultured neurons have shown that perfusing these cells with N-terminal tau fragments leads to an increased release of beta amyloid, which can be blocked by N-terminal tau antibodies [23]. A better understanding of the biological mechanisms underpinning the interaction between amyloid and tau pathologies may well progress in concert with the development of combination therapies and benefit from the growing knowledge of the impact of these therapies on measurable biomarkers [24].

Another argument for the potential benefit of anti-amyloid and tau combination therapy is the limited impact of anti-amyloid antibodies [3], when administered later in the disease course, on measurable biomarkers of tau pathology, which are closely correlated to cognitive decline [25]. One possibility is that at these stages, significant neuronal damage has already occurred, leading to a relatively modest impact on mitigating cognitive decline. Alternatively, insoluble tau accumulation in lysosomes may impair cellular mechanisms necessary to realize the maximal benefit of amyloid clearance [26]. In this context, timely targeting of both pathways, either sequentially or simultaneously, may offer the best chance for therapeutic benefit.

In addition to the advantages and disadvantages of the different study designs, as described above, there are several specific considerations for combining anti-amyloid and anti-tau treatments. The simultaneous use of anti-amyloid and anti-tau therapies could offer the best chance for synergistic effects, providing early benefits from both, but may be associated with a higher risk of adverse events. The initiation of anti-tau treatment, following amyloid clearance, can mitigate the risk of adverse events of the anti-amyloid agent but also postpone the potential benefits associated with tau removal. Alternatively, if it is assumed that tau seeding is the primary driver of cognitive decline and occurs early in the disease course, it may be reasonable to initiate anti-tau therapy first and discontinue it earlier, particularly if it proves to be highly effective. Anti-amyloid therapy could be then introduced. In conclusion, despite a strong scientific rationale for their association, the optimal strategy for combining anti-amyloid and anti-tau therapies has yet to be determined.

Beyond the potential use of anti-amyloid and anti-tau agents simultaneously, the combination of other categories of therapeutics might also be productive. Other combinations can be envisioned, including drugs targeting neuroinflammation, cell metabolism and bioenergetics, oxidative stress, or synaptic plasticity [27,28].

### 4.4 What is the role of biomarkers in combination clinical trials?

Remarkable developments in biofluid and imaging markers of AD neuropathological changes have led to the incorporation of these biomarkers into biological staging criteria, alongside new clinical staging schemas [29]. Biomarkers are now routinely integrated into clinical trials to confirm the presence of AD neuropathological changes (as inclusion criteria), to assess the biological staging of the disease, and to measure the target engagement of interventions. In combination clinical trials, establishing baseline biomarker status prior to each intervention is crucial for determining whether the combination therapy alters neuropathology and whether it impacts disease progression more effectively than a single agent alone. Thus, biomarkers are becoming key outcome measures in combination clinical trials as they serve as objective measures to monitor biological changes in disease progression [2,3,30] and to help determine whether the trial intervention successfully modifies the physiopathology of AD, providing evidence of the treatment's effectiveness. The implementation of biomarkers for neuronal damage, synaptic function, and inflammation will also be essential. Given that synapse damage and loss are fundamental to the pathophysiology of AD and lead to reduced cognitive function, synaptic biomarkers will ultimately play a critical role in assessing the bottom-line effects of combination therapies, in addition to biomarkers measuring the effects or target engagement of the individual therapies [31].

The use of biomarkers in clinical trials is more complex in the setting of combination therapies. For example, anti-amyloid monoclonal antibodies have been shown to reduce p-tau 181, p-tau 217, and glial fibrillary acidic protein (GFAP) in concert with plaque reduction [32,33]. This may compromise the ability to use these markers to interrogate the effects of combination therapies. It will be important to understand the impact of each agent on biomarkers and to identify biomarkers that may be unique to each component of the combination [7].

## 5 Conclusions and perspectives

Task Force recommendations converge on the conclusion that new combination therapies for AD are both urgent and necessary. Combination trials represent a pivotal step toward transformative therapeutic advancements in AD. The combination therapy model is supported by the complex pathogenesis of AD and the limitations of the clinical benefit of monotherapy alone. The goal of combination therapy should be to ensure patient safety while optimizing the likelihood of treatment success. Firstly, the design of AD clinical trials should be tailored to minimize the risk of adverse events (e.g., ARIAs). Optimizing the likelihood of success requires the identification of potential responders' profiles. Biomarker characterization and responder analysis from clinical trials data have the potential to identify populations most likely to benefit from each specific combination. The key lesson learned from the development of anti-HIV combination therapy is that once the underlying biology is understood, effective solutions can be rapidly developed. Optimizing the likelihood of success primarily requires a deeper understanding of the precise mechanisms underpinning disease development, especially those involved in the interactions between amyloid and tau pathology. Combination therapy has been a standard practice in the field of oncology for several years, offering valuable insights that can be applied to the field of AD. One key lesson from oncology is the potential of repurposed drugs, including previously discontinued therapies, which, when used in combination, may lead to more effective treatments. Another important lesson is the potential benefits of adaptive trial designs, as these flexible approaches allow researchers to modify ongoing trials based on emerging data, thereby improving efficiency and success rates.

The concept of combination therapy is not entirely new in the field of AD. Indeed, formulations combining acetylcholinesterase inhibitors and memantine are already commercialized, and background therapies with these drugs are commonly allowed in contemporary clinical trials. However, the development of combination therapies involving disease-modifying agents marks a new chapter in the AD drugs development pipeline, that is expected to expand in the near future. There are currently 126 drugs in the pipeline and choosing the best combinations, among these and/or repurposed drugs approved for other indications, will be a major challenge. Computational approaches could provide valuable insights. The goal is to select drugs that overlap extensively with the disease pathogenesis, while minimizing overlap between them to reduce the risk of synergistic side effects. The development of multi-target drugs is an integral part of combination therapies and can help reduce some of the operational complexities. The advent of anti-amyloid treatments in clinical practice may be an opportunity to pave the way for the widespread use of combination therapies in real-world settings. Platforms, including public-private partnerships, must be established to streamline the implementation of combination therapies and address major issues (e.g., financial, regulatory, operational) that could hinder their development.

## Declaration of AI assistance in writing

AI-assisted technologies were utilized in the creation of this document. Specifically, AI tools were employed for figure structuring. While AI contributed to the drafting process, the authors conducted all final edits, fact-checking, and refinements to ensure accuracy, originality, and alignment with the intended objectives.

## Declaration of competing interest

The Task Force was partially funded by registration fees from industrial participants. These corporations placed no restrictions on this work. **D.A** is an investigator in clinical trials sponsored by Alector, Alzheon, Acadia, Aribio, Biogen, Eisai, Genentech, GSK, Green Valley, Hoffmann-La Roche, Janssen, Medesis Pharma, Nestlé, Novo Nordisk, Otsuka, Regenlife, UCB Pharma. He received consulting fees from Novo Nordisk and lecture fees from Eisai. **L.M** has received research funding from Johnson & Johnson, Merck, Takeda, Eisai, Gates Ventures, the Davos Alzheimer's Collaborative, UKRI and ADDF, all to Institution. **R.B** has received research support from the National Institutes of Health, the National Institute on Aging, Alzheimer's Association, GHR Foundation, an anonymous organization and the DIAN-TU Pharma Consortium, Biogen, Eisai, Eli Lilly and Company/Avid Radiopharmaceuticals, F. Hoffman-La Roche/Genentech, and Janssen. He has also received funding from Eisai, Avid Radiopharmaceuticals, Janssen, Cogstate, Cerveau, and Signant Health, AbbVie, Bristol Myers Squibb, and Novartis. RJB co-founded C2N Diagnostics, which offers the PrecivityAD2 blood test. Washington University and RJB have equity ownership interest in C2N Diagnostics and receive income based on technology and receives income from C2N Diagnostics for serving on the scientific advisory board. He has served as an unpaid member of advisory boards for Roche and Biogen. **P.A.** has a research collaboration with Eisai and consults with Lilly, Merck, Roche, Genentech, Abbvie, Biogen, ImmunoBrain Checkpoint, AltPep, Alector, Arrowhead and Neurimmune. **A.B** has served as a paid consultant to Alector, Alexion, Arrowhead, Arvinas, Eli Lilly, Merck, Neurocrine, Ono, Oscotec, Switch and Transposon. Institution received research support from Biogen and Eisai for serving as a site investigator for clinical trials, as well as from Regeneron. **S.S.** served as consultant and/or Advisor for Cognition Therapeutics, Eisai Inc, Guidepoint global, Expert Connect; and received Grant/Research Support from: Aribio, Biogen, Cognition Therapeutics, Eisai Inc., Eli Lilly, Janssen. **J. Z.,** is a fully time employee of Eisai Inc. **I. G**. is a full time employee and stockholder of F. Hoffmann-La Roche. **R.R.** has received research support from Eli Lilly and Eisai as part of public-private partnership grant, all to the Institution. **S.Sa.** reports grants and personal fees from Lilly, Roche, Biogen, Genentech, Eisai, Acumen; personal fees from NovoNordisk, Neurophet, Acumen, Prothena, LabCorp, Abbvie, outside the submitted work. He is an Associate Editor of the Journal of Prevention of Alzheimer's and of Alzheimer's and Dementia: Diagnosis, Assessment and Disease Monitoring. **H. F.** reports personal fees from Alector, Promis, outside the submitted work. **R.S.** reports personal fees from Abbvie, AC Immune, Acumen, Alector, Apellis, Biohaven, Bristol Myers Squibb, Genentech, Janssen, Nervgen, Oligomerix, Prothena, Roche, Vigil Neuroscience, Ionis, Vaxxinity, grants and other from Alzheimer's Association, grants from National Institute on Aging, GHR Foundation, Eli Lilly, Eisai, other from Clinical Trials in Alzheimer's Disease, outside the submitted work. **B. V.** is the Founding President of IHU HealthAge (ANR-23-IAHU-0011), affiliated with Toulouse University Hospital and Inserm Cerpop. He is also an investigator in clinical trials sponsored by several industry partners. In the past three years, he has served as a Scientific Advisory Board (SAB) member for Biogen, Alzheon, Novo Nordisk, Lilly, Eisai, and Roche, without any personal compensation. **J.C.** has provided consultation to Acadia, Acumen, ALZpath, Annovis, Aprinoia, Artery, Axsome, Biogen, Biohaven, BioXcel, Bristol-Myers Squib, Cervomed, Eisai, Fosun, GAP Foundation, Green Valley, IGC, Janssen, Kinoxis, Lighthouse, Lilly, Lundbeck, LSP/eqt, Mangrove Therapeutics, Merck, MoCA Cognition, New Amsterdam, Novo Nordisk, NSC Therapeutics, Optoceutics, Otsuka, Oxford Brain Diagnostics, Praxis, Prothena, ReMYND, Roche, Scottish Brain Sciences, Signant Health, Simcere, Sinaptica, T-Neuro, TrueBinding, and Vaxxinity pharmaceutical, assessment, and investment companies. He has stocks/options in Artery, Vaxxinity, Behrens, Alzheon, MedAvante-Prophase, Acumen. He is supported by NIGMS grant P20GM109025; NINDS grant U01NS093334; NIA grant R01AG053798; NIA grant P30AG072959; NIA grant R35AG71476; NIA R25 AG083721–01; Alzheimer's Disease Drug Discovery Foundation (ADDF); Ted and Maria Quirk Endowment; Joy Chambers-Grundy Endowment. **J.C.** owns the copyright to the Neuropsychiatric Inventory (NPI).
